# Medication discrepancies among hospitalized patients with hypertension: assessment of prevalence and risk factors

**DOI:** 10.1186/s12913-021-07349-5

**Published:** 2021-12-14

**Authors:** Rana Abu Farha, Alaa Yousef, Lobna Gharaibeh, Waed Alkhalaileh, Tareq Mukattash, Eman Alefishat

**Affiliations:** 1 Department of Clinical Pharmacy and Therapeutics, Faculty of Pharmacy, Applied Science Private University, Amman, Jordan; 2 Faculty of Medicine, Al Balqa’ Applied University, Salt, Jordan; 3 Pharmacological and Diagnostic Research Center, Faculty of Pharmacy, Al-Ahliyya Amman University, Amman, Jordan; 4 Department Biopharmaceutics and Clinical Pharmacy, Faculty of Pharmacy, The University of Jordan, Amman, Jordan; 5 Department of Clinical Pharmacy, Faculty of pharmacy, Jordan University of Science and Technology, Irbid, Jordan; 6 Department of Pharmacology, College of Medicine and Health Science, Khalifa University of Science and Technology, P O Box 127788, Abu Dhabi, United Arab Emirates; 7 Center for Biotechnology, Khalifa University of Science and Technology, Abu Dhabi, United Arab Emirates

**Keywords:** Medication errors, Hypertension, Hospital, Assessment, Jordan

## Abstract

**Background:**

Medication errors remained among the top 10 leading causes of death worldwide. Furthermore, a high percentage of medication errors are classified as medication discrepancies. This study aimed to identify and quantify the different types of unintentional medication discrepancies among hospitalized hypertensive patients; it also explored the predictors of unintentional medication discrepancies among this cohort of patients.

**Methods:**

This was a prospective observational study undertaken in a large teaching hospital. A convenience sample of adult patients, taking ≥4 regular medications, with a prior history of treated hypertension admitted to a medical or surgical ward were recruited. The best possible medication histories were obtained by hospital pharmacists using at least two information sources. These histories were compared to the admission medication orders to identify any possible unintentional discrepancies. These discrepancies were classified based on their severity. Finally, the different predictors affecting unintentional discrepancies occurrence were recognized.

**Results:**

A high rate of unintentional medication discrepancies has been found, with approximately 46.7% of the patients had at least one unintentional discrepancy. Regression analysis showed that for every one year of increased age, the number of unintentional discrepancies per patient increased by 0.172 (P = 0.007), and for every additional medication taken prior to hospital admission, the number of discrepancies increased by 0.258 (P= 0.003). While for every additional medication at hospital admission, the number of discrepancies decreased by 0.288 (P < 0.001). Cardiovascular medications, such as diuretics and beta-blockers, were associated with the highest rates of unintentional discrepancies in our study. Medication omission was the most common type of the identified discrepancies, with approximately 46.1% of the identified discrepancies were related to omission. Regarding the clinical significance of the identified discrepancies, around two-third of them were of moderate to high significance (n= 124, 64.2%), which had the potential to cause moderate or severe worsening of the patient´s medical condition.

**Conclusions:**

Unintentional medication discrepancies are highly prevalent among hypertensive patients. Medication omission was the most commonly encountered discrepancy type. Health institutions should implement appropriate and effective tools and strategies to reduce these medication discrepancies and enhance patient safety at different care transitions. Further studies are needed to assess whether such discrepancies might affect blood pressure control in hypertensive patients.

## Introduction

Despite multidisciplinary efforts and collaborations to ensure patient safety, medication errors remained among the top 10 leading causes of death worldwide [1]. Medication discrepancies are among the most commonly encountered medication errors, which occurred in up to 70% of hospitalized patients [2]. Medication discrepancies are defined as unexplained changes among regimens across different care sites [3]. They are classified as unintentional and intentional discrepancies, and the latter sub-classified into documented or undocumented [4, 5]. Intentional documented discrepancies are not errors, while undocumented intentional discrepancies were noted down as documentation errors. Unintentional discrepancies are considered medication errors that need to be prevented or resolved. They are classified into several categories: omission of a required drug previously used, the addition of a medication not previously used and not justified by the patient’s clinical condition, duplication of medications, dosage discrepancies, frequency discrepancies, administration route discrepancies, or dosage form discrepancies [4, 5].

Discrepancies are considered a health problem that may lead to harmful clinical and economic consequences such as interruptions of treatment, inadequate prescriptions, adverse drug events, increased hospital readmissions, and the duration of hospitalization, which will increase the medical cost [4]. One of the most critical risk factors that could increase the susceptibility and frequency of medication discrepancies is the lack of a validated system for medication reconciliation [6].

The Institute for Healthcare Improvement (IHI) defined medication reconciliation as “the process of creating the most accurate list possible of all medications a patient is taking including medication name, dosage, frequency, and route and comparing that list against the physician’s admission, transfer, and/or discharge orders, intended to provide correct medications to the patient at all transition points within the hospital” [7]. This process was successful and effective in detecting and preventing most discrepancies from reaching the patients [5, 8]. Additionally, it was effective in improving medication adherence and decreasing hospital readmission [9]. Based on the Health Care Institute resources and facilities, medication reconciliation intervention could be done effectively using several methods, including using the standardized form, electronic reconciliation tools, pharmacy-led programs, and collaborative models between health care professionals [10]. Furthermore, according to reports from the third Global Patient Safety Challenge, namely Medication Without Harm, which was established by World Health Organization (WHO), reducing medication errors and improving patient safety should focus upon three priority groups; those transitioning between settings of care, higher risk patients and those receiving polypharmacy [11].

Hypertensive patients are a group of patients who are susceptible to recurrent medication changes and poly-drug regimens, therefore, they are particularly vulnerable to medication errors, including discrepancies in their medications [12]. Thus, this study aimed to identify and quantify the different types of unintentional discrepancies among hospitalized hypertensive patients; it also explored the predictors of unintentional discrepancies among this cohort of patients.

## Methods

### Clinical setting, study design, and participants

This prospective cross-sectional observational study recruited hypertensive patients admitted to the internal medicine and general surgery departments at Jordan University Hospital (JUH), the largest tertiary teaching hospital in Jordan. Patients were eligible for inclusion if they fulfilled the following inclusion criteria: (1) aged ≥18 years, (2) previously diagnosed with hypertension (diagnosis was confirmed from patients’ medical record), (3) having an intended length of stay of more than 24 h, (4) using a minimum of four regular medications before admission, (5) Arabic speaking, and (6) agreed to sign the study consent form.

Patients were excluded from the study if they (1) were isolated due to infectious diseases or compromised immunity, (2) cognitively disabled with no caregiver, or (3) unwilling to provide written informed consent.

### Data collection

The study researcher was available for patients’ recruitments from 9 am to 1 pm, five days a week (from Sunday to Thursday) during the period from November 2017 to January 2019 (15-months interval). A convenience sample of patients’ medical records was screened and reviewed to check patients’ eligibility. All eligible patients who agreed to participate in the study were asked to sign a written informed consent and were then interviewed during their hospital stay by a well-trained clinical pharmacist to collect all required data using a pre-prepared data collection form. The pharmacist received qualified training that involved data collection, identifying and resolving medication discrepancies in a standardized and systematic manner.

Data collected included date of admission, chief compliant, intended length of stay, other comorbid medical conditions, medications prescribed at the admission date (including medication name, dose, frequency, route of administration, dosage form, start and stop dates), and length of hospital stay.

Information regarding patients’ medications taken prior to admission, denoted by the Best Possible Medication History (BPMH), was also collected. To achieve optimal BPMH, information regarding prescription medications, non-prescription medications (Over the Counter) like supplements and herbal preparations, recreational medications, and as-needed medications were collected [13]. Information included medication name, dose, frequency, route of administration, dosage form, and duration of treatment. Patients’ BPMH was collected from two different sources, including the patient’s medication sheet (within the medical record), and the patient/patient’s caregiver interview. During these patient or patient’s caregiver interviews, patients were asked about their medications, their names, the dosage regimen they follow, and whether their medications or a medication list were available with them in hospital. In the case that patients or caregivers were unable to recall any requested information, caregivers were asked to either bring all the medications taken by the patient at home during their next visit or send us pictures of the actual box or list of medications.

### Identifying medication discrepancies

After obtaining the BPMH, a comparison between the BPMH list and the admission medication order was carried out to detect the presence of any discrepancies. The comparison process further consisted of examining every medication on the BPMH list and comparing it with the admission medication order. When dealing with combination products, each component was considered as a single medication. Medication discrepancies are classified as unintentional and intentional discrepancies, and the latter are sub-classified into documented or undocumented. Intentional documented discrepancies are “clinically understandable and appropriate discrepancies between the BPMH and the admission medication order based on the patient’s plan of care” [13]. While intentional undocumented discrepancy is “one in which the prescriber has made an intentional choice to add, change or discontinue a medication but this choice is not documented” [13]. The last type includes unintentional discrepancy which is “one in which the prescriber unintentionally changed, added or omitted a medication the patient was taking prior to admission” [13].

During the comparison, any discrepancy that was documented in the patient’s medical file was termed as an intentional documented discrepancy, such as adding antibiotics to treat acute conditions (e.g., infections). If the discrepancy was undocumented, then the pharmacist discusses the discrepancy with the physician to verify if the changes were intentional or made by error. In the case where the physician made the change intentionally, then it was recorded as an “undocumented intentional discrepancy”. Otherwise, it was considered an “unintentional discrepancy”.

Undocumented intentional discrepancies were noted down as documentation errors. Unintentional discrepancies were considered medication errors, those included: omission of a required medication previously used, the addition of a medication not previously used and not justified by the patient’s clinical condition, duplication of medications, dosage discrepancies, frequency discrepancies, administration route discrepancies, and dosage form discrepancies.

Finally, unintentional discrepancies were classified into three classes based on the level of their seriousness as described by Cornish et al. [14]. In this classification, unintentional discrepancies were categorized into class I if they were unlikely to cause patient discomfort or clinical deterioration. While classes II and III included unintentional discrepancies that could cause moderate or severe discomfort or clinical deterioration, respectively [14].

Unintentional discrepancies were classified based on their clinical seriousness by the authors of the study, i.e., based on subjective assessment, and in the case of disagreement on classification, the seriousness of the discrepancy was discussed until consensus was reached.

### Sample size calculation

The sample size was calculated based on the number of subjects per predictor needed to conduct linear regression analysis as recommended by Tabachnick and Fidell (5-20 subjects per predictor) (Tabachnick and Fidell, 2006). Using 20 subjects per predictor, and since we have eight predictors, a minimum sample size of 160 was considered to be representative.

### Statistical analysis

Data were entered and analyzed using Statistical Package for the Social Sciences (SPSS) version 22 (SPSS Inc., Chicago, IL, USA). The descriptive analysis was done using median and interquartile range (IQR), and frequency and percentages for categorical variables.

Linear regression was carried out to initially screen the independent variables that affect the number of identified unintentional discrepancies. Variables found to have p value< 0.25 using univariate linear regression analysis were entered into multivariate linear regression analysis. Variables were selected after checking their independence, where tolerance values > 0.2 and Variance Inflation Factor values were < 5 indicate the absence of multicollinearity between the independent variables in regression analysis. In the multivariate linear regression analysis, variables that were independently affecting the number of identified unintentional discrepancies were identified. A p value of ≤0.05 was considered statistically significant.

## Results

During the study period, a total of 382 patients were screened for their eligibility criteria; of these, two patients were excluded since they were less than 18 years, 57 patients had less than four medications, and 64 patients do not have hypertension. Two hundred fifty-nine patients were eligible and agreed to participate in the study (response rate 100%). Patients had a median age of 66.0 years (IQR=15.0), and males represented 52.9% of the study sample (n = 137). The majority of patients were married (80.7%, n = 209). Approximately 50% of patients had primary school or high school degrees (n = 122, 47.1%), and 60.2% had a monthly income of less than 250 JD per month (Table 1).

**Table 1:** Socio-demographic characteristics of the study sample (n= 259)

Parameter	Median (IQR)	n (%)
Age, years	66.0 (15.0)	
Gender		
Males		137 (52.9)
Females		122 (47.1)
Marital Status		
Single		8 (3.1)
Married		209 (80.7)
Divorced		3 (1.2)
Widowed		39 (15.1)
Educational level		
Not educated		43 (16.6)
Primary School/high school		122 (47.1)
Diploma/BSc		87 (33.6)
Masters/PhD		4 (1.6)
Monthly Income^a^		
≤ 250 JD		156 (60.2)
251-500 JD		43 (16.6)
501-750JD		47 (18.1)
751-1000 JD		8 (3.1)
> 1000 JD		4 (1.5)

^a^1 JD= 0.71 US$, *IQR *interquartile range

The median number of medical conditions for the study patients was 3.0 (IQR = 2.0), while the median number of medications taken prior to admission and admission medications were 7.0 (IQR= 4.0) and 9.0 (IQR= 5.0), respectively. Approximately 60% of the patients (n = 157, 60.6%) were admitted to the internal medicine department, while the rest were admitted to the surgery department (Table 2).

**Table 2:** Medical histories and administrative data of the study sample (n= 259)

Parameter	Median (IQR)	n (%)
Number of medical conditions	3.0 (2.0)	
Number of medications taken prior to admission	7.0 (4.0)	
Number of admission medications	9.0 (5.0)	
Length of Stay (days)	5.0 (6.0)	
Admission department		
Internal medicine		157 (60.6)
Surgery		102 (39.4)

*IQR *interquartile range

**Fig. 1:**
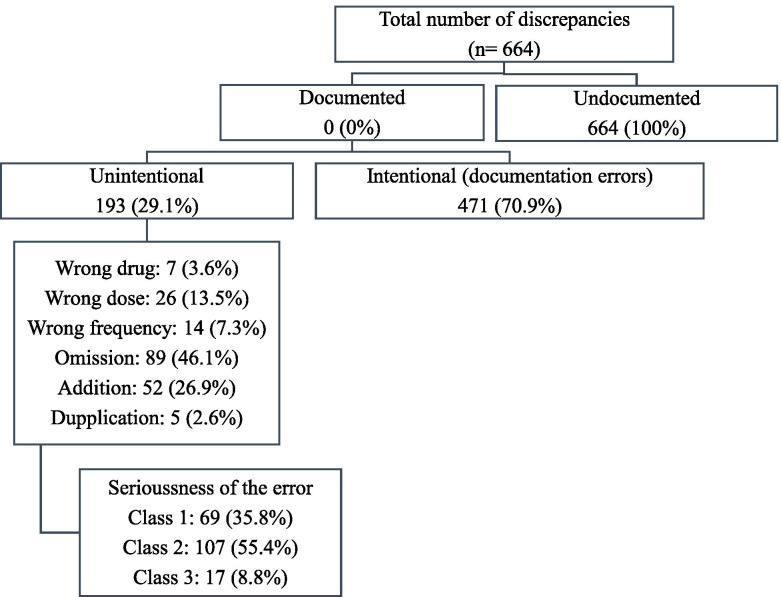
Classifications of medication discrepancies identified among study sample

During the study period, a total number of 664 undocumented discrepancies were found. Of those, 471 (70.9%) discrepancies were intentional (errors in the documentation), while 29.1% of them were unintentional (n = 193). The unintentional discrepancies were further classified, in which omission was the most commonly found (n = 89, 46.1%), followed by the addition of new medications (n = 52, 26.9%). Examples of such discrepancies were “the omission of enalapril for a patient without any justification”, and “the addition of atorvastatin that was discontinued previously due to myopathy”, respectively. Regarding the seriousness of the identified unintentional discrepancies, only 8.8% of the discrepancies (n = 17) were classified as class 3 (severely serious discrepancies), while 55.4% of them (n= 107) were classified as class 2 (moderately serious discrepancies). For more details, refer to Fig. 1.

**Fig. 2:**
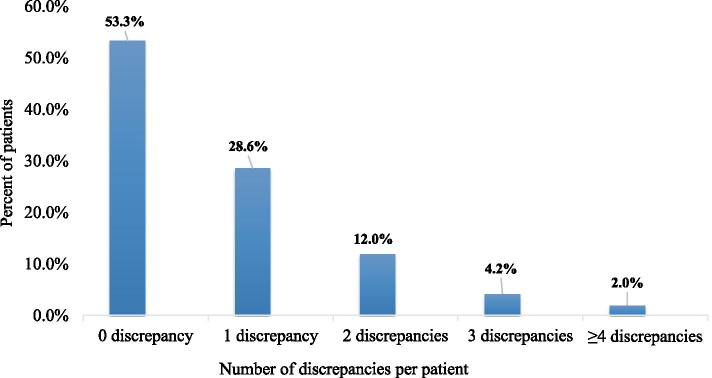
The distribution of patients based on the number of unintentional discrepancies they experienced (n= 259)

Patients were distributed based on the number of unintentional discrepancies they experienced, and as seen in Fig. 2, approximately 46.7% of the participants (n = 121) were found to have at least one unintentional discrepancy, while 53.3% (n= 138) had no discrepancies at all).

Unintentional discrepancies most commonly involved cardiovascular medications such as diuretics and beta-blockers with a percentage of 30.1% (n = 58), whereas gastrointestinal medications such as proton pump inhibitors, and H2 blockers come in the second place (n = 51, 26.4%). Oncology related medications were the least commonly involved in medication discrepancies (n= 1, 0.5%) (Table 3).

**Table 3:** Distribution of unintentional medication discrepancies based on drug classification (n= 193)

Types of medications	Number of unintentional discrepancies per medication category (%)
Cardiovascular medications	58 (30.1)
Oncology related medications	1 (0.5)
Neurology related medications	7 (3.6)
Gastrointestinal medications	51 (26.4)
Endocrine related medications	24 (12.4)
Rheumatology related medications	3 (1.6)
Vitamins and supplements	22 (11.4)
Others	27 (14.0)

The effect of different variables on the number of unintentional discrepancies (Table 4) showed that for every one year of increased age, the number of unintentional discrepancies per patient increased by 0.172 (p value= 0.007), and for every additional medication taken prior to hospital admission, the number of discrepancies increased by 0.258 (p value= 0.003). While for every additional medication taken at hospital admission, the number of discrepancies decreased by 0.288 (p value<0.001).

**Table 4:** Regression analysis for risk factors affecting the number of unintentional discrepancies among study sample (n = 259)

Variables	Dependent variable Number of unintentional discrepancies			
	Univariate linear regression		Multivariate linear regression	
	Beta	p value	Beta	p value
Age (years)	0.191	0.002^a^	0.172	0.007^b^
Gender (1: males, 2: females)	0.129	0.038^a^	0.099	0.137
Educational level	-0.121	0.053^a^	-0.003	0.963
Monthly Income	-0.071	0.255	-	-
Number of Medical Conditions	0.158	0.011^a^	0.104	0.113
Number of medications taken prior to admission	0.094	0.130^a^	0.258	0.003^b^
Number of Admission Medications	-0.113	0.070^a^	-0.288	<0.001^b^
Length of Stay (days)	-0.017	0.784	-	-

^a^ Eligible for entry in multivariate linear regression, ^b^Significant at 0.05 level. Beta: standardized regression coefficient

## Discussion

This prospective cross-sectional observational research was conducted to identify and quantify the different types of unintentional medication discrepancies among hospitalized hypertensive patients admitted to a medical or surgical ward in a large teaching hospital in Jordan. The findings of this study showed a high rate of unintentional medication discrepancies among this cohort of patients, with approximately 47% of the patients had at least one unintentional discrepancy. Medication omission is the most commonly encountered discrepancy type.

Comparing various studies that evaluated reconciliation errors and discrepancies is hindered by differences in the patients’ characteristics, methodology, clinical setting, and specific interpretation of discrepancies. However, each study provides an insight into the prevalence of these medication errors in that setting and population. Medication errors can be assessed at different junctures of medical care; at admission, during the transition from one level to another in the hospital, at discharge, and during follow-up [15, 16]. Ashcroft et al. prospective study showed that medication errors were more likely to develop at hospital admission, with an odds ratio of 1.70 (95% CI 1.61-1.80), than during the patients’ hospital stay [17]. This finding demonstrates the importance of evaluating medication reconciliation at hospital admission.

The average age of our patients was 65 years; the study included patients with hypertension, a condition that is more prevalent among the elderly. The age of patients in other studies that examined reconciliation at admission varied from having younger [15] and older participants [18, 19].

In our study, a high rate of unintentional medication discrepancies was found among hypertensive patients; with at least one discrepancy in 47% of patients. This finding is close to those reported in other similar studies where the rate of identified discrepancies ranged from 33.2 to 53.6% [14, 20], but it is still less than that revealed by one study that was conducted in an internal medicine ward in Switzerland where there was at least one discrepancy for every patient [19]. Most of the discrepancies identified in this study involved cardiovascular medications such as diuretics and beta-blockers. This result is in concordance with data revealed by other studies, where cardiovascular medications were the medications most often involved in discrepancies [21, 22].

Various risk factors were evaluated as predictors for the occurrence of the unintentional discrepancies. Our study revealed that age is a statistically significant factor (P = 0.007), which is in agreement with risk factors uncovered in several studies [21, 23, 24]. Polypharmacy demonstrated an interesting effect on the number of discrepancies, depending on the patients’ pre-admission or admission medication order. An increase in the number of medications taken prior to admission led to a significant increase in the number of discrepancies; similar results identified this critical link between the number of medications taken prior to admission and the risk of errors at hospital admission [18, 25]. Patients, who are medically managed with numerous medications, is highly susceptible to medication-related problems upon admission to the hospital [26].

The number of admission medications (those prescribed upon hospital admission) was associated with a decrease in the number of the identified discrepancies. One possible explanation is that health care providers, especially physicians, scrutinize in more details hospitalized patients with co-morbidities and multiple medications for errors and potential medication-related problems.

Comparable to other studies [18, 20], medication omissions were the most common discrepancy with a frequency of 46.1%. This type of error possesses severe consequences since it deprives the patient of treatment for a specific medical condition at the hospital, where more focused and extensive patient care is expected.

Regarding the clinical seriousness of the identified discrepancies, more than 64% of them had the potential to cause moderate or severe worsening of the patient´s medical condition. This finding was higher than the percentage reported by studies conducted in Canada (38.6%), France (27.2%), and USA (11.7%) which reported a lower percentages of discrepancies with serious clinical impact [14, 20, 23].

This study demonstrated the need to implement effective tools and strategies to reduce discrepancies and enhance patient safety at different transitions of care, among them is to implement the reconciliation process. The implementation of the reconciliation process, especially at hospital admission or discharge, was effective in reducing medication errors as proved by previous literature [4, 5, 27].

Our study has several limitations that need to be pointed out. Initially, this study was conducted in a single teaching hospital, a multi-centered study would give a more comprehensive idea of the prevalence of medication discrepancies upon hospital admission. Another limitation is that hypertensive patients were only recruited from two wards (the internal medicine and general surgery departments); investigation of discrepancies at admission in other medical wards will provide a more thorough assessment.

Moreover, the impact of discrepancies on patients’ different clinical outcomes, such as the effect on blood pressure levels, was not investigated. The effect on blood pressure levels were not assessed, since patients with hypertension may be anxious upon admission, which could affect their blood pressure reading upon admission. Also, discrepancies identified upon admission may need time to have an impact on blood pressure, and this can be only evaluated through longitudinal studies. In addition, the number of medical conditions that were counted and studied as a possible predictor for the occurrence of discrepancies were based on the total number of medical conditions, rather than counting those related to hypertension. Finally, the assessment of the seriousness of the unintentional medication discrepancies was conducted on discrepancies that were identified at the admission date, and we did not followed-up patients or reviewed their records to determine if discrepancies were corrected, which may affect their level of seriousness.

## Conclusions

Unintentional medication discrepancies are highly prevalent among hypertensive patients. Medications used for the management of cardiovascular diseases are highly liable to medication errors, especially the omission of medications previously used by the patient. Health institutions should implement appropriate and effective tools and strategies to reduce these discrepancies and enhance patient safety at different transitions of care. Further studies are needed to assess whether such discrepancies might affect blood pressure control in hypertensive patients.

## Data Availability

All data generated or analyzed during this study are included in this published article.

## Abbreviations

BPMH: Best Possible Medication History
SPSS: Statistical Package for Social Science
